# Lability of IgE Levels Early in Life

**DOI:** 10.1155/2011/547389

**Published:** 2011-06-20

**Authors:** Koffi N'guessan, David Ternant, François Labarthe, Hervé Watier

**Affiliations:** ^1^Laboratoire d'Immunologie Faculté de Médecine, 10 Boulevard Tonnellé, 37032 Tours Cedex, France; ^2^Pilot Center for Therapeutic Antibody Monitoring, CHRU de Tours, France; ^3^Université François-Rabelais de Tours, France; ^4^CNRS, UMR6239, France; ^5^Faculté de Médecine d'Abidjan, Cote D'Ivoire; ^6^Laboratories of Pharmacology, CHRU de Tours, France; ^7^Pediatric Department, CHRU de Tours, France; ^8^INSERM, U921, France

## Abstract

We report a case of a very fast and intriguing decrease in IgE concentrations after exclusion from the diet of any CM lysate in an unusual clinical presentation of cow's milk allergy in an infant. Analysis of IgE kinetics after allergen elimination suggests rapid cessation of IgE biosynthesis and a short IgE half-life.

## 1. Introduction

An unusual clinical presentation of cow's milk allergy (CMA) in an infant gave us the opportunity to observe a very fast and intriguing decrease of IgE concentrations after allergen withdrawal, raising new questions about IgE production and metabolism in newborns and infants. 

## 2. Case Report

**Figure 1:**
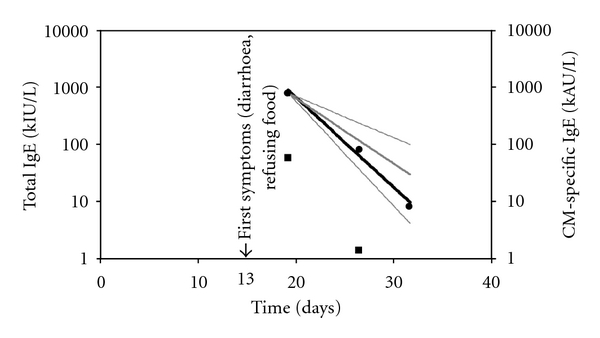
Evolution of total IgE (●) and CM-specific IgE (■). IgE levels were measured on D19, D26, and D31. Predicted IgE kinetics are indicated as a black bold line. Extrapolated IgE kinetics are shown as grey lines (median in bold, 5 and 95% CI as normal lines). With only two values available, calculations were not performed for specific IgE.

A 17-day-old neonate, born to atopic parents at full term, was hospitalized in the children hospital of Tours (France) for poor feeding and increasing diarrhoea for 4 days, associated with a severe metabolic acidosis, after a symptom-free interval of 2 weeks. The diarrhoea ceased soon after admission. Erythema and a pustular rash of the face as well as a “gloves and socks-” type skin rash were noted at 18 days of age, leading to the finding of very high concentrations of total IgE (1298 kIU/L) and IgE specific to cow's milk (CM) (83 kAU/L, Phadia) (Figure 1). First-stage formula milk had been introduced on day 11 in addition to breastfeeding according to the mother, although it is possible that this had been given since birth. Although the clinical presentation was very unusual and severe [1, 2], the diagnosis of CMA was confirmed by a rapid regression of all symptoms after withdrawal of CM proteins. Seven days later, total IgE fell to 121 kIU/L and specific IgE to 1.44 kAU/L, and total IgE returned to within the normal range (10.5 kIU/L) (Figure 1) five days later. 

## 3. Discussion

The IgE detected in the newborn had been self-produced since IgE did not cross the placenta and his mother had no CM-specific IgE (not shown). This IgE immune response thus probably reflected a rapid maturation of IgE+ B cells into plasmablasts, as recently evidenced in mice [3]. No less spectacular was the 100-fold decrease in IgE concentrations 12 days later (Figure 1), following exclusion of any CM lysate from the diet. High levels of both specific and total IgE are regularly observed in infant cow's milk allergy [4] and are reminiscent of experimental models where their coexistence reflects a genetic (atopic) background [5]. However, their parallel evolution, and the fact that they both appeared to be strictly antigen dependent, could also suggest that the IgE measured in the “total” assay is mostly directed against CM.

Pharmacokinetic modelling techniques were used to describe the elimination kinetics of total IgE in this infant. The kinetics model was ln (*C*(*t*)) = ln (*C*_n_) − *k*_*e*_ · *t*, where *C*(*t*) is the IgE concentration over time (kIU/L), *C*_*n*_ is the estimated IgE concentration just after CM withdrawal, *k*_*e*_ (day^−1^) is the first-order elimination constant, and *t* (day) is time. The *k*_*e*_ parameter was estimated using log-linear regression. The elimination half-life (*T*_1/2 _) was calculated as *T*_1/2_ = ln (2)/*k*_*e*_ and was 1.7 days in this infant. The combined kinetics of IgE and omalizumab, an anti-IgE humanized monoclonal antibody, were previously described in adults using PK-PD modelling [6]. The parameters describing the interindividual distribution of IgE elimination kinetics in this study were used to estimate the confidence interval containing 90% of *T*_1/2_ values (CI90_T_1/2__), which was (1.5–3.8) days. Although low, the *T*_1/2_ of IgE of our infant was within this CI90_T_1/2__ and thus has to be considered as “normal.” However, to calculate this elimination kinetics, it was postulated that IgE production ceased after CM withdrawal, which is not necessarily the case. It is therefore possible that the true IgE *T*_1/2_ of this patient may be shorter than 1.7 days.

Nevertheless, our findings suggest that the IgE production stopped rapidly—if not immediately—after allergen elimination, suggesting that a very limited IgE plasmablast life span combined with the very short IgE half-life. Whether this hypothetical IgE plasmablast short life span is restricted to IgE or whether it is due a more general immaturity of lymphoid niches in the newborn, as suggested in murine models [7], warrants further investigations in a cohort of CM allergic newborns.
